# Mozambique’s response to cyclone Idai: how collaboration and surveillance with water, sanitation and hygiene (WASH) interventions were used to control a cholera epidemic

**DOI:** 10.1186/s40249-020-00692-5

**Published:** 2020-06-16

**Authors:** Joaquim Domingos Lequechane, Arlete Mahumane, Falume Chale, Crescêncio Nhabomba, Cristolde Salomão, Clemente Lameira, Sérgio Chicumbe, Cynthia Semá Baltazar

**Affiliations:** 1 Beira Operational Research Center, National Institute of Health from Mozambique, Beira, Sofala Mozambique; 2 National Institute of Health, Maputo, Mozambique; 3 Planning and Statistics Department, Sofala Provincial Health Directorate, Beira, Mozambique

**Keywords:** Cyclone Idai, Mozambique, Cholera, Sanitation, Disaster, Disease outbreak, Water supply

## Abstract

Cyclone Idai, which hit Mozambique in March 2019, was one of the worst climate-related natural disasters on record in the Southern Hemisphere causing massive destruction of housing and disruption to vital infrastructure including the electrical grid, communications and water supply. Almost two million people were affected with over 600 deaths, hundreds of thousands of people displaced accompanied by rapid spread of cholera. We describe emergency measures taken by the Government of Mozambique, in collaboration with multilateral partners, to establish a real-time disease surveillance system, implement interventions recommended by a Water, Sanitation and Hygiene (WASH) taskforce and rapidly scale up a massive community vaccination program to control a cholera epidemic.

## Cyclone Idai – a massive weather disaster in Mozambique

Mozambique ranks among the top three African countries most vulnerable to weather-related events, since the cyclones events in 2019. Cyclone Idai, a category 4 cyclone, struck Mozambique on 14 March 2019 affecting five provinces with heavy rains, severe flooding, and devastating winds [1]. Nearly 1.85 million people were affected with an official death toll of 603. The cyclone completely destroyed approximately 122 700 houses and partially destroyed another 111 200 [2] along with severe damage to 77 health facilities [3]. The transportation, infrastructure, water supply, electric grid and communications services were damaged, interrupting commercial activity as well as access for rescue services [1, 3]. Around 400 000 displaced people were accommodated in shelters, most with poor access to basic services [2].

In response, the President of Mozambique declared a State of Emergency on 19 March 2019 and the World Health Organization (WHO) declared the humanitarian situation, Grade 3 on 25 March 2019 under the WHO Emergency Response Framework [2].

The Government of the Republic of Mozambique (GRM), through the National Institute of Disaster Management (INGC), coordinated the emergency humanitarian response. The response was supported by a Humanitarian Country Team (HCT) composed of United Nations agencies, donors, international and local non-governmental organizations (NGOs) and other partners. A health cluster (a coalition including 42 partners) working predominantly in Sofala Province was activated in support of the GRM led response [1, 3, 4]. The health cluster, led by WHO, included partners such as Médecins Sans Frontières (MSF), United Nations Office for the Coordination of Humanitarian Affairs (UNOCHA), United Nations Children’s Fund (UNICEF), International Committee of the Red Cross (ICRC), United Nations World Food Programme (WFP), International Organization for Migration (OIM), Medicus del Mundo, World Vision, Samaritan’s Purse and others [5]. These various partners came together to collaborate on various aspects of the response effort including health, nutrition, water, sanitation and hygiene (WASH), protection, shelter, food security, education and others.

A WASH cluster was established and was led by UNICEF to develop a strategy to ensure the provision of safe water, sanitation and hygiene facilities for the people in shelters and those residing in the path of the cyclone with the goal of reducing the risk of transmission of WASH related preventable disease [6].

## Cholera outbreak following the natural disaster

As a result, the heavy rains and flooding severely impacted sanitation and access to safe drinking water in the most affected areas. Most of the localities had their homes destroyed and latrines overflowed thus contaminating the water supply. Overcrowded conditions in emergency rescue shelters were further compromised by the limited access to safe water. The level of infrastructure destruction caused by Idai, combined with the high level of contaminated water sources, poor sanitation and living conditions led to a rapid spread of infectious diseases and a cholera outbreak that was officially declared on 27 March 2019 [2]. By 21 April 2019, 6768 suspected cholera cases were registered with an attack rate of 571 per 100 000 habitants, and 8 deaths (case fatality rate: 0.12%) [7]. The most affected districts were Beira, Dondo, Nhamatanda and Búzi in Sofala Province; Beira is the largest district in Sofala Province (533 825 habitants), followed by Nhamatanda (317 538 habitants). These districts are densely populated where the majority of the population already live in extreme poverty with limited access to safe drinking water [8].

## Using real time surveillance data to guide WASH interventions

As part of the humanitarian response, an emergency taskforce and surveillance system was established by the Ministry of Health (MoH) through the National Institute of Health (INS) to ensure early detection and response to any outbreak of infectious diseases in the most affected districts as a result of the impact of the cyclone. In the initial phase, a paper-based surveillance system was established that prioritized identification of four clinical features or diseases including fever, diarrhea, cholera and malaria. The system rapidly transitioned to an online *Early Warning Alert and Response System (EWARS).*

EWARS is a simple robust and cost-effective tool developed by the WHO to improve disease outbreak detection and response during emergencies including political conflicts and natural disasters. The mobile and web-based applications include automated analysis and alert modules able to trigger signals of an outbreak at an early stage. The system is also capable of generating automated reports and bulletins throughout the outbreak response gathered from event-based and indicator-based surveillance. Data can be collected offline and then synchronized with the online EWARS application when a connection is available [4]. The system allowed reporting by age group of diagnosed diseases or undiagnosed signs/symptoms like cholera, fever, diarrhea, malaria, malnutrition, measles and pellagra but also events such as rumors of suspected outbreaks elsewhere in the community. Rumors and alerts generally rely upon syndromic case definitions which results in a lower threshold for rumor reporting. But, because its goal is to trigger rapid response teams, it can report a case as suspect, and sometimes confirmed, just based on symptoms and signs alone.

EWARS was rolled out covering 67 health facilities in the 4 most affected districts: Buzi, Dondo, Nhamatanda and Beira City. The data was sent daily by the Surveillance Officer from each health facility to Emergency Operation Center, however, when an alert was triggered, an email was sent to the national, provincial and district level surveillance officers where an alert manager at the provincial level would immediately make contact with the reporting health facility for confirmation or disposal of the alert.

Due to the emphasis on the notifiable conditions to be reported in EWARS, other disease programs expressed concerns that routine communicable disease surveillance efforts could temporarily discarded, and historical trends could be missed. To address this concern, efforts were made to clarify that EWARS is specifically designed to support rapid notification and detection during the emergency situation. In comparison, routine surveillance systems rely on several variables (symptoms, signs and laboratory diagnosis) before confirming a condition. As a result, it does not interfere with nor be regarded as substituting the routine surveillance reporting systems.

## Collaboration and partnership using data as a guide for action

A Cholera Taskforce was established to integrate the WASH team and health cluster stakeholders. During the coordination meetings, detailed surveillance data produced by EWARS was shared allowing rapid implementation of measures to prevent and treat cholera and deploy environmental management resources to provide clean and safe water where urgently needed. WASH activities included chlorination of drinking water, providing supplies for household water treatment, regular monitoring of drinking water (residual chlorine), providing health facilities with essential commodities (Oral Rehydration Solution - ORS, cholera kits, high test hypochlorite and others), and other supplies for infection control and patient care, intensification of hygiene promotion activities, and other related activities [5, 6].

After the emergency declaration, GRM and partners acted swiftly to contain the cholera outbreak establishing multiple oral rehydration points (OPRs), Cholera Treatment Units (CTUs) and Cholera Treatment Centers (CTCs) in partnership with MSF and Red Cross support [9, 10]. UNICEF worked with partners to build latrines and boreholes/wells in resettlement sites as existing boreholes/wells in the affected areas were systematically rehabilitated and disinfected. WASH teams decontaminated and disinfected clothes from residences of cholera patients and suspects when discharged from health facilities and provided chlorine for home use.

A cholera diagnostic testing algorithm was implemented to ensure (i) monitoring the progress toward control of the outbreak and (ii) document the cholera strains involved. All fecal samples collected from the CTUs and CTCs were tested using a one-step rapid diagnostic test (Crystal® VC, RDT). All positive samples were sent in less than 6 h to the provincial laboratory for culture (TCBS and Mueller-Hinton culture medium). Isolates of cholera confirmed cases were sent to the INS Microbiology Laboratory (in Mueller-Hinton culture medium for up to 72 h) for molecular confirmation and additional testing.

The integration of laboratory diagnostic testing with the surveillance data combined with real time data analysis not only improved patient clinical management but also enabled the implementation of a communication strategy that provided rapid and accurate information disseminated through daily and weekly epidemiological bulletins.

## Real-time surveillance to inform the implementation of oral cholera vaccination (OCV) in emergency setting

The surveillance data was also crucial for the mapping of high-risk areas which were targeted for an oral cholera vaccination (OCV) campaign. From 3 to 9 April 2019, an OCV mass vaccination campaign was conducted in the four most affected districts in Sofala Province that targeted more than 800 000 individuals older than 1 year of age. The overall coverage achieved was 98.6% of the target population. In the two-week period after the OCV campaign and the reinforced WASH activities, a significant decrease in reported suspect cholera cases was observed across all four of the most affected districts. Strong government leadership and collaboration between the surveillance and WASH team, combined with an effective OCV campaign, was able to rapidly contain the cholera outbreak in Sofala Province.

## Conclusions and recommendation

Lessons learned from Cyclone Idai demonstrated the importance of strong government leadership and collaborative multisectoral engagement on the immediate action that linked real-time surveillance data to the WASH interventions and vaccination campaign to respond to a cholera outbreak.

First, it is important that governments have a comprehensive emergency response plan that is updated regularly to maintain a resilient health system and prevent cholera outbreaks in future similar events. Investments in the health system and public WASH strategies are crucial. In addition, prevention and control of diarrheal diseases, including cholera in complex emergencies required the development of a National Cholera Plan (NCP). Next, the engagement of multisectoral stakeholders and partners is essential to leverage resources for maximum coverage and impact. Stakeholders must also include disease-specific technical working teams and the health workforce. Finally, EWARS implementation was a success because it directed surveillance resources to priority disease conditions and produced real-time actionable data that allowed for targeted investigation and response.

In conclusion, the Cyclone Idai emergency provided an opportunity for Mozambique to improve its surveillance systems and preparedness strategies for future emergencies, using a multi-sectoral collaboration approach.

## Data Availability

Not applicable.

## Abbreviations

CTC: Cholera Treatment Center
CTU: Cholera Treatment Units
EWARS: Early Warning Alert and Response System
GRM: Government of the Republic of Mozambique
HCT: Humanitarian Country Team
ICR: International Committee of the Red Cross
INGC: Mozambique National Institute of Disaster Management
INS: Mozambique National Institute of Health
MoH: Ministry of Health
MSF: Médecins Sans Frontières
NCP: National Cholera Plan
NGOs: Non-governmental organizations
OCV: Oral cholera vaccination
OIM: International Organization for Migration
ORS: Oral Rehydration Solution
RDT: Rapid Diagnostic Test
UNICEF: United Nations Children’s Fund
UNOCHA: United Nations Office for the Coordination of Humanitarian Affairs
WASH: Water, Sanitation and Hygiene
WFP: United Nations World Food Programme
WHO: World Health Organization
